# Inhibition of IKKα by BAY61-3606 Reveals IKKα-Dependent Histone H3 Phosphorylation in Human Cytomegalovirus Infected Cells

**DOI:** 10.1371/journal.pone.0150339

**Published:** 2016-03-01

**Authors:** Catherine M. K. Ho, I’ah Z. Donovan-Banfield, Li Tan, Tinghu Zhang, Nathanael S. Gray, Blair L. Strang

**Affiliations:** 1 Institute of Infection & Immunity, St George’s, University of London, Cranmer Terrace, London, SW17 0RE, United Kingdom; 2 Department of Cancer Biology, Dana-Farber Cancer Institute, Boston, MA 02115, United States of America; 3 Department of Biological Chemistry & Molecular Pharmacology, Harvard Medical School, Longwood Ave, Boston, MA 02115, United States of America; University of St Andrews, UNITED KINGDOM

## Abstract

Protein kinase inhibitors can be used as tools to identify proteins and pathways required for virus replication. Using virus replication assays and western blotting we found that the widely used protein kinase inhibitor BAY61-3606 inhibits replication of human cytomegalovirus (HCMV) strain AD169 and the accumulation of HCMV immediate-early proteins in AD169 infected cells, but has no effect on replication of HCMV strain Merlin. Using *in vitro* kinase assays we found that BAY61-3606 is a potent inhibitor of the cellular kinase IKKα. Infection of cells treated with siRNA targeting *IKKα* indicated IKKα was required for efficient AD169 replication and immediate-early protein production. We hypothesized that IKKα was required for AD169 immediate-early protein production as part of the canonical NF-κB signaling pathway. However, although BAY61-3606 inhibited phosphorylation of the IKKα substrate IκBα, we found no canonical or non-canonical NF-κB signaling in AD169 infected cells. Rather, we observed that treatment of cells with BAY61-3606 or siRNA targeting *IKKα* decreased phosphorylation of histone H3 at serine 10 (H3S10p) in western blotting assays. Furthermore, we found treatment of cells with BAY61-3606, but not siRNA targeting *IKKα*, inhibited the accumulation of histone H3 acetylation (H3K9ac, H3K18ac and H3K27ac) and tri-methylation (H3K27me3 and H3K36me3) modifications. Therefore, the requirement for IKKα in HCMV replication was strain-dependent and during replication of an HCMV strain requiring IKKα, IKKα-dependent H3S10 phosphorylation was associated with efficient HCMV replication and immediate-early protein production. Plus, inhibition of HCMV replication by BAY61-3606 is associated with acetylation and tri-methylation modifications of histone H3 that do not involve IKKα.

## Introduction

Cellular protein kinases are required for many aspects of viral replication and pathogenesis. During human cytomegalovirus (HCMV) replication cellular proteins kinases have prominent roles in genome replication as intracellular signaling pathways that contain protein kinases have been implicated in activation of viral and cellular transcription during productive replication [1].

Productive HCMV transcription from the viral genome first requires activation of the HCMV immediate early promoter (MIEP), which controls production of the immediate-early viral proteins IE1 and IE2 [1]. These proteins stimulate a transcriptional cascade of immediate-early to early to late RNA transcripts required for production of infectious virus [1]. The MIEP is a complex promoter that can be recognized by transcription factors controlled by intracellular signaling pathways containing cellular protein kinases [1]. Perhaps the least understood, and most controversial, signaling pathway reported to be involved viral transcription is the canonical NF-κB signaling pathway.

During canonical NF-κB signaling [2] phosphorylation of serines in the IKKα/β heterodimer (IKKα-P(Ser176/180)) leads to phosphorylation of the repressor protein IκBα at serine 32 (IκBα-P(Ser32)) by IKKα. Phosphorylation of IκBα triggers its proteasomal degradation. Degradation of IκBα allows the release of a transcriptional activator heterodimer (RelA/p50) bound to IκBα, which translocates from the cytoplasm to the nucleus and activates transcription. RelA in the RelA/p50 heterodimer can be phosphorylated by a number of cellular kinase proteins, which can dictate its function. This includes phosphorylation of RelA serine 536 (RelA-P(Ser536)) by kinases such as IKKα, IKKβ and IκBα [2,3]. Non-canonical NF-κB signaling, which also requires IKKα, has also been described [2]. This pathway is dependent on the proteasomal processing of the repressor protein p100 to p52, which is stimulated by phosphorylation of p100 by IKKα. The absence of p100 allows the translocation of transcription factor heterodimer RelB/p52 from the cytoplasm to the nucleus to activate transcription.

The role of canonical NF-κB signaling during productive HCMV replication is unclear. It has been reported that deletion of NF-κB responsive elements in the MIEP has no effect on productive HCMV replication [4] and canonical NF-κB signaling is not required for productive HCMV replication [5]. Conversely, it has also been reported that canonical NF-κB signaling is required for transactivation of the MIEP [6], that there is up regulation of canonical NF-κB responsive promoters in HCMV infected cells [7,8] and inhibition of canonical NF-κB signaling impairs HCMV replication [6,9,10]. The role of canonical NF-κB signaling in activation of cellular transcription during productive HCMV replication is also unclear, but it has been reported that canonical NF-κB signaling is associated with transcription of cellular genes involved in productive HCMV replication [11,12]. At least one previous report indicates that non-canonical NF-κB signaling does not occur during productive HCMV replication [13].

Another factor in the control of HCMV gene expression is transcriptional regulation dictated by histone proteins. This, also, is not well understood. During productive HCMV replication histones associate with the viral genome [14]. It has been reported that histone acetylation is required for efficient viral gene transcription [14,15] and modifications of histone H3 most commonly associated with active transcription, (acetylation of H3 at lysine 9 and lysine 14 (H3K9ac and H3K14ac, respectively)), are found at viral promoters, including the MIEP, throughout productive HCMV replication [14]. The presence of these acetylation modifications is likely to be involved in the recruitment of transcription factors to chromatin.

The full catalogue of modifications that are present on histone H3 during productive HCMV replication has yet to be determined. However, it is likely that phosphorylation of H3 occurs in HCMV infected cells as evidence from studies of uninfected cells points to a link between histone phosphorylation and acetylation. For example, H3 phosphorylation at residue serine 10 (H3S10p) is related to the presence of H3K14ac on H3 [16] and there is a functional relationship between the presence of H3S10p, H3K9ac and H3K14ac and the recruitment of transcription factors to chromatin [17]. Interestingly, phosphorylation of H3 can be mediated by IKKα [16,18–21], a protein kinase involved in both canonical and non-canonical NF-κB signaling.

We sought to expand our understanding of intracellular signaling required for productive HCMV replication. Previously, data from one of our laboratories using protein kinase inhibitor BAY61-3606 suggested that germinal center kinase (GCK, also known as MAP4K2) can function upstream of NF-κB signaling [22]. It was unknown if GCK can affect NF-κB signaling in HCMV infected cells. Therefore, we examined HCMV replication in the presence of BAY61-3606.

## Materials & Methods

### Drugs

BAY61-3606 [23] and related compounds [22] were synthesized by the Gray laboratory or obtained from SIGMA (UK). GCK inhibitors 5, 16, 17 have been previously described [24] and were synthesized by the Gray laboratory. Maribavir [25] was a kind gift from John Drach (University of Michigan). All drugs were resuspended in dimethyl sulphoxide (DMSO). Tumor necrosis factor alpha (TNF-α) and cycloheximide were kind gifts from Steve Goodbourn (St George’s, University of London).

### Cells and viruses

Human foreskin fibroblast (HFF) cells (clone Hs29) were obtained from American Type Culture Collection no. CRL-1684 (ATCC, Manassas, VA)). B cell line HB7 (an lymphoblastoid cell line established by infection of adult B cells with the BAC-cloned B95-8 strain of EBV[26]) was a kind gift from Robert White (Imperial College London) and lymphocyte cell line Jurkat was obtained from the Dana-Farber Cancer Center, Boston, USA. All cells were maintained in Dulbeccos Modified Eagles Medium (DMEM) (Gibco) containing 5% fetal bovine serum (FBS) (Gibco), plus penicillin and streptomycin. High passage HCMV strain AD169 was a gift from Don Coen (Harvard Medical School) and low passage strain Merlin(RCMV1111) (derived from BACmid pAL1111, which does not express RL13 and UL128 [27]) was gift from Richard Stanton (Cardiff University).

### Viral yield reduction assays

Assays were performed essentially as described in [28]. HFF cells were plated at a density of 5 × 10^4^ cells per well in 24-well plates. After overnight incubation, cells were infected with HCMV at a multiplicity of infection (MOI) of 1. After virus adsorption for 1 hr at 37°C, cells were washed and incubated with 1ml of media containing DMSO or BAY61-3606 at concentrations indicated in the text. Plates were incubated for 96 hours at 37°C. Titers were determined by serial dilution of viral supernatant onto HFF monolayers which were covered in DMEM media containing 5% FBS and 0.6% methylcellulose. Cultures were incubated for 14 days, cells were stained with crystal violet and plaques were counted. All drug concentrations were tested in duplicate. The final concentration of DMSO in all samples was maintained at <1% (v/v).

### Plaque reduction assays

Assays were performed essentially as described in [28]. HFF cells were seeded at a density of 2 × 10^5^ cells per well in 24-well plates. After 24 hours incubation, cells were infected with HCMV at 40 PFU per well in DMEM plus 5% FBS at 37°C. One hour post-infection, the inocula were removed, cells were washed, and media containing various concentrations of DMSO or BAY61-3606, 5% FBS and 0.6% methylcellulose were added. All drug concentrations were tested in duplicate. After incubation at 37°C for 14 days, cell monolayers were stained with crystal violet and plaques were counted. The final concentration of DMSO in all samples was maintained at <1% (v/v).

### Cytotoxicity assays

Assays were performed essentially as described in [28]. HFF cells were seeded at a density of 1 × 10^4^ cells per well into 96-well plates. After 4 hours incubation to allow cell attachment, cells were treated for the time indicated in the text with different concentrations of each compound in duplicate. Cell viability was then determined with an MTT assay (GE Healthcare) according to the manufacturer's protocol. The final concentration of DMSO in all samples was maintained at <1% (v/v). As a positive control, in all experiments a 2-fold dilution series of HFF cells starting at 1 × 10^4^ cells per well was included. In each experiment we found a linear relationship between the number of cells per well and output from the MTT assay (data not shown).

### Viral infection of HFF cells and treatment with drugs for Western blotting analysis

5 x 10^4^ or 1 x 10^5^ HFF cells per well were seeded in 24- or 12-well plates, respectively, 24 hours before infection. At the time of infection virus was added to each well at the MOI indicated in the text. After incubation for 1 hour at 37°C, virus supernatant was removed and replaced with 1 ml of complete Dulbeccos Modified Eagles Medium (DMEM) (Gibco) containing 5% fetal bovine serum (FBS) (Gibco) containing either DMSO or drugs at the indicated concentrations. At indicated time points viral supernatant was titred onto HFF monolayers and/or cells were washed once with PBS and resuspended in 100 μl Laemmli buffer containing 5% β-mercaptoethanol.

### Western blotting

Lysate from 5 x 10^3^ or 1 x 10^4^ cells was used to detect cellular and viral antigens, respectively. Western blotting of proteins separated on 8% or 10% polyacrylamide gels was carried out as described elsewhere [29], using antibodies recognizing IE1/2, UL44, pp28, UL84 (all Virusys, 1:1000 dilution), IE2 proteins (clone 5A8.2, Millipore, 1:1000 dilution), β-actin (SIGMA, 1:5000 dilution), SYK (ab3993) and GCK (ab167532 or ab184169) (all Abcam, 1:1000 dilution). Antibodies recognizing UL112-113 proteins [30] or UL97 [31] were kind gifts from Shang-Kwei Wang (Kaohsiung Medical University, Taiwan) and Donald Coen (Harvard Medical School, USA), respectively. All antibodies recognizing proteins involved in NF-κB signaling or H3 proteins were obtained from Cell Signaling Technology (products #9936, #4888, #9927, #9847, #9783) and used as per the suppliers instructions. All primary antibodies were detected using anti-mouse- or anti-rabbit-horseradish peroxidase (HRP) conjugated antibodies (Millipore and Cell Signaling Technology, respectively). Chemiluminescence solution (GE Healthcare) was used in each case to detect secondary antibodies using film. The intensity of certain bands detected by western blotting was assessed using Photoshop CS4. To compare band intensity, the mean intensity from an identical area around each band was found and subtracted from a “blank” area on the same blot. Data shown is the band intensity normalized to the β-actin controls shown in each lane of that figure expressed as arbitrary units (a.u.).

### Transfection of siRNA into HFF cells

Briefly, 1 x 10^5^ HFF per well were seeded in 12-well plates 24 hours before transfection in DMEM+5%FBS with no antibiotics. siControl Non targeting siRNA #3, ON-TARGETplus SMARTpool Human GCK or ON-TARGETplus SMARTpool Human IKKα (all Dharmacon) were used. Per well, 113 μl of 1 μM siRNA and 2 μl Dharmafect2 (Dharmacon) were diluted in 93 μl and 146 μl Optimem (Invitrogen), respectively. After 5 mins at room temperature, both solutions were combined. After 20 mins, media was removed from each well and replaced with the siRNA/Dharmafect mixture then 500 μl of DMEM+5%FBS with no antibiotics was added to each well. Transfected cells were incubated at 37°C for 72 hours then either prepared for western blotting or infected as indicated in the text.

To ensure that IKKα siRNAs do not non-specifically bind to either viral or cellular mRNAs, interaction of each siRNA from the IKKα siRNA pool was analyzed using online genome-wide enrichment of seed sequence mapping (GESS) (http://www.flyrnai.org/gess/). We found no obvious off-target binding of any IKKα siRNA to any cellular mRNA or any mRNA transcript expressed from the AD169 genome (data not shown).

### *In vitro* analysis of kinase activity

All assays were conducted using the KinaseProfiler™ service Eurofins Pharma Discovery Services UK Limited. Briefly, recombinant protein kinases were purified from baculovirus cells and purified by affinity chromatography using the proteins tags mentioned below. Each kinase was resuspended in 50 mM TRIS, 0.1 mM EGTA, 0.1 mM Na3VO4, 0.1% β-mercaptoethanol, 1 mg/mL BSA (SYK, LYN) or 20 mM MOPS, 1 mM EDTA, 0.01% Brij-35, 5% Glycerol, 0.1% β-mercaptoethanol, 1 mg/mL BSA (GCK, IKKα, IKKβ). In each reaction;

1. SYK. Full length His-tagged protein was used. Kinase was incubated with 50 mM Tris pH 7.5, 0.1 mM EGTA, 0.1 mM Na3VO4, 0.1% β-mercaptoethanol, 0.1 mg/ mL poly(Glu, Tyr) 4:1, 10 mM MgAcetate and [γ-33P-ATP].
2. GCK. Residues 1–473 glutathione-s-transferase (GST) tagged protein was used. Kinase was incubated with 8 mM MOPS pH 7.0, 200 mM NaCl, 0.2 mM EDTA, 0.8 mg/mL MBP, 10 mM MgAcetate and [γ-33P-ATP].
3. IKKα. Full length GST-tagged protein was used. Kinase was incubated with 8 mM MOPS pH 7.0, 0.2 mM EDTA, 200 μM peptide, 10 mM MgAcetate and [γ-33P-ATP].
4. IKKβ. Full length His-tagged protein was used. Kinase was incubated with 8 mM MOPS pH 7.0, 0.2 mM EDTA, 100 μM peptide, 10 mM MgAcetate and [γ-33P-ATP].
5. Lyn. Full length His-tagged protein was used. Kinase was incubated with 50 mM Tris pH 7.5, 0.1 mM EGTA, 0.1 mM Na3VO4, 0.1% β-mercaptoethanol, 0.1 mg/mL poly(Glu, Tyr) 4:1, 10 mM MgAcetate and [γ-33P-ATP].

In each reaction the specific activity of [γ-33P-ATP] was approximately 500 cpm/pmol. Each reaction was initiated with the addition of 10 μM MgATP. After incubation for 40 minutes at room temperature, reactions were stopped with the addition of 3% phosphoric acid. Ten μL of the reaction is then spotted onto Filtermat A or P30 filtermat and washed three times for 5 minutes in 75 mM phosphoric acid and once in methanol prior to drying and scintillation counting. As indicated in the text and Figure Legends, in each reaction 10 μM BAY61-3606 or the equivalent volume of DMSO was added to reactions containing each protein kinase. To determine IC50 concentrations, a range of BAY61-3606 concentrations (100–0.01 μM) or the equivalent volumes of DMSO were added to reactions containing IKKα. IC50 data was analyzed using XLFit version 5.3 (ID Business Solutions). To calculate IC50 values sigmoidal dose-response (variable slope) curves were fitted using non-linear regression analysis.

## Results

### Inhibition of HCMV replication and immediate-early protein production by BAY61-3606

We employed viral yield reduction and viral plaque reduction assays to assess the ability of BAY61-3606 to inhibit replication of HCMV strain AD169 in human foreskin fibroblast (HFF) cells. AD169 is a high passage HCMV strain that has previously been used to study nearly all aspects of HCMV replication [32]. In both assays we found 50% Effective Dose and 90% Effective Dose (ED50 and ED90, respectively) values in the range of 0.2–1.2 μM (Table 1). These values are similar to those for inhibition of HCMV replication by the frontline therapy drug ganciclovir [28,33], indicating BAY61-3606 is an effective inhibitor of HCMV replication. To exclude the possibility that the observed reduction in HCMV replication is due to BAY61-3606 toxicity in HFF cells, we exposed HFF cells to BAY61-3606 at a range of concentrations and used an MTT dye-uptake assay to assess cell viability. This assay indicated that BAY61-3606 had a 50% Cytotoxicity Concentration (CC50) value of greater than 100 μM (Table 1). Thus, the ability of BAY61-3606 to inhibit AD169 replication is unlikely to be due to drug toxicity in HFF cells.

**Table 1:** Viral inhibition and cytotoxicity assays using BAY61-3606.

BAY61-3606				
Assay	Viral Strain	ED50^1^	ED90^1^	CC50^1^
Viral Yield Reduction^2^	AD169	0.5	1.2	-
Viral Yield Reduction^2^	Merlin(RCMV1111)	>10	>10	-
Viral Plaque Reduction^2^	AD169	0.2	0.4	-
MTT Cytotoxicity^3^	-	-	-	>100

^1^ μM

^2^Viral titre was assessed at 96 hours infection in the presence of drug.

^3^MTT assays were carried out after 96 exposure of cells to drug.

We also used the viral yield reduction assay to asses the ability of BAY61-3606 to inhibit replication of HCMV strain Merlin(RCMV1111) [27] in HFF cells. Merlin is low passage strain of HCMV whose genomic content is more similar to wild type HCMV than high passage HCMV strains such as AD169 [32]. We found that BAY61-3606 did not obviously inhibit Merlin(RCMV1111) replication at concentrations up to 10μM (Table 1). Therefore, the ability of BAY61-3606 to inhibit HCMV replication differs between strains of HCMV.

**Fig 1:**
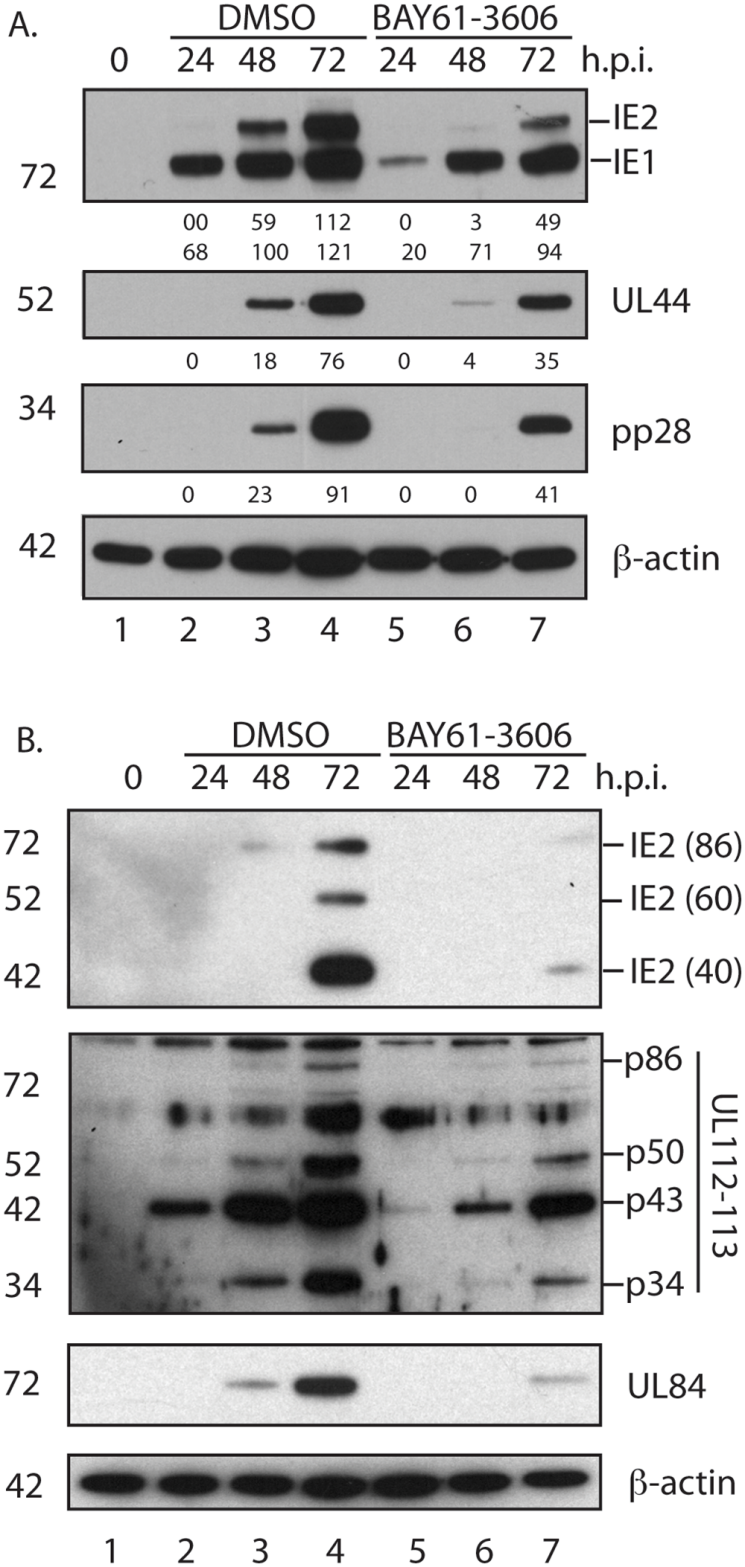
Analysis of viral proteins in HCMV infected HFF cells treated with DMSO or BAY61-3606. (A and B) HFF cells were uninfected or infected with AD169 at an MOI of 1, then treated with either 1μM BAY61-3606 or the equivalent volume of DMSO. Cell lysates were prepared for western blotting at the time points (hours post infection (h.p.i.)) indicated above the figures. Uninfected cells harvested at the time of infection are shown as 0 h.p.i.. Proteins recognized by the antibodies used in each experiment are indicated to the right of each figure. The positions of molecular weight markers (kDa) are indicated to the left of each figure. In Fig 1A the band intensities are expressed in arbitrary units below each panel.

To investigate how BAY61-3606 inhibits AD169 replication we used western blotting to analyze the presence of immediate-early (IE1/IE2), early (UL44) and late (pp28) viral proteins in HFF cells infected with AD169 and treated with either DMSO or BAY61-3606 (Fig 1). We observed a 2- to 3-fold reduction in the accumulation of IE1, IE2, UL44 and pp28 in AD169 infected cells treated with BAY61-3606 at 72 h.p.i. (Fig 1A, lanes 5–7), compared to those treated with DMSO (Fig 1A, lanes 2–4). In this and subsequent western blotting experiments the amount of *β*-actin in each sample was also assayed to demonstrate equivalent loading of samples in each lane. We hypothesized that BAY61-3606 reduces accumulation of the immediate-early proteins IE1 and IE2, which leads to a reduction in UL44 and pp28. This was supported by further western blotting experiments (Fig 1B). Compared to treatment of AD169 infected cells with DMSO (Fig 1B, lanes 2–4), the treatment of infected cells with BAY61-3606 (Fig 1B, lanes 5–7) inhibited the accumulation of IE2 (IE2-86) and IE2 proteins that are expressed late in replication (IE2-60 and IE2-40 [34]), plus the accumulation of proteins produced from the viral *UL112-113* (p84, p50, p43, p34) locus whose expression is dependent on transcriptional activation by IE2 [35] and viral protein UL84, whose post-translational stability requires the presence of IE2 [36,37]. In each case a 2- to 4-fold decrease was found by analyzing band intensity, except for IE2 proteins, which showed an over 5-fold decrease at 72 h.p.i. (data not shown). Therefore, treatment of AD169 infected HFF cells with BAY61-3606 results in inhibition of viral immediate early protein accumulation. Furthermore, treatment of AD169 infected cells with BAY61-3606 at the time of infection results in a dramatic decrease in viral replication (Table 1), but a more modest decrease in immediate-early protein production (Fig 1A and 1B). Therefore, inhibition of viral replication by BAY61-3606 is likely to inhibit the production or function of viral or cellular factors required for productive replication other than viral immediate-early proteins.

### Potential BAY61-3606 targets in HCMV infected cells

**Fig 2:**
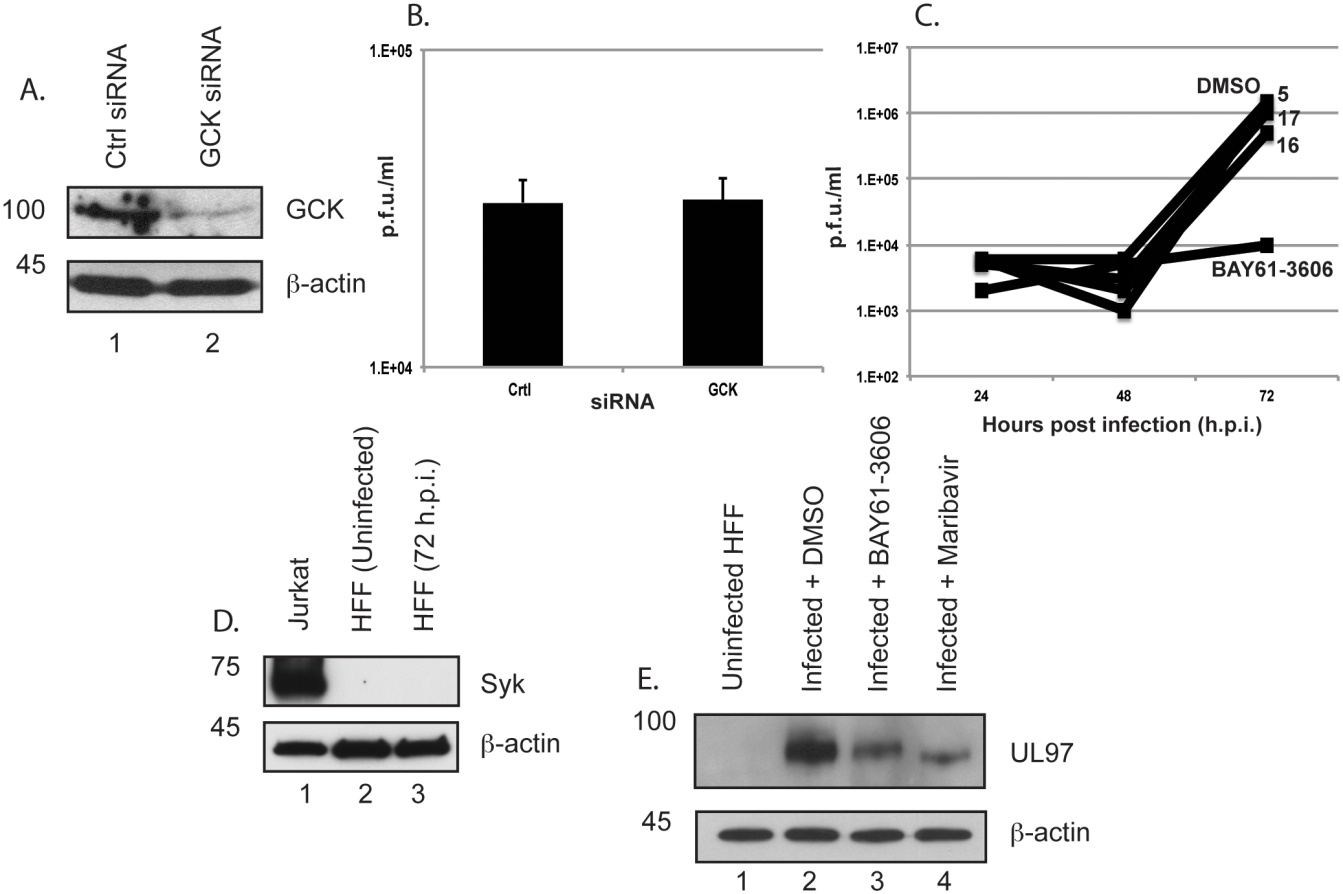
Investigation of roles for SYK and GCK in HCMV infected cells. (A and B) Analysis of siRNA treated cells infected with AD169. HFF were treated with the siRNAs indicated in each figure for 72 hours and infected with 1x10^5^ plaque forming units (p.f.u.) of HCMV. At 96 h.p.i cell lysates were prepared for western blotting (Fig 2A) and viral supernatants were harvested for virus titration (Fig 2B). In Fig 2B viral titre is expressed as plaque forming units/ml (p.f.u./ml) and the mean and standard deviation of 3 experiments is shown. (C) Analysis of HCMV infected cells treated with inhibitors of GCK. HFF cells were infected with AD169 at an MOI of 1 then treated with 1μM of the drug indicated in the figure or the equivalent volume of DMSO. Viral supernatants were harvested at the indicated time points and viral titre (p.f.u./ml) at each time point was determined. (D and E) Western blotting of lysate from uninfected or infected cell lines. Unless stated otherwise, all lysates are from HFF cells. Where indicated, HFF cells were either uninfected or infected with AD169 at an MOI of 1 then treated with either 1μM BAY61-3606, 1μM Maribavir or the equivalent volume of DMSO. Cell lysates were prepared for western blotting at 72 hours post infection. In each panel showing western blotting proteins recognized by the antibodies used in each experiment are indicated to the right of each figure and the positions of molecular weight markers (kDa) are indicated to the left of each figure.

We hypothesized that GCK is a target of BAY61-3606 in HFF cells infected with AD169. To assess if GCK is required for productive HCMV replication we treated HFF cells with either siRNA targeting *GCK* mRNA (GCK siRNA) or a control siRNA that has no target in human cells (Ctrl siRNA). We then infected siRNA treated cells with AD169 and either used western blotting to assess GCK protein levels or assayed production of HCMV (Fig 2A and 2B, respectively). While we observed a reduction in GCK in infected cells treated with GCK siRNA (Fig 2A, lane 2) compared to Ctrl siRNA (Fig 2A, lane 1), we found no difference in production of HCMV from HFF cells treated with either Ctrl siRNA or GCK siRNA (Fig 2B). Furthermore, we also treated AD169 infected HFF cells with either potent and specific inhibitors of GCK structurally unrelated to BAY61-3606 (compounds 5, 16 and 17 [24]), BAY61-3606 or DMSO and found that only BAY61-3606 inhibited AD169 replication (Fig 2C). Therefore, as removal or inhibition of GCK had no effect on productive AD169 replication it is unlikely GCK is the target of BAY61-3606 in this context.

BAY61-3606 has been reported to inhibit the cellular kinase protein spleen tyrosine kinase (SYK) [23]. Using western blotting (Fig 2D) we could detect SYK in the lymphocyte cell line Jurkat (Fig 2D, lane 1), but not in either uninfected or AD169 infected HFF cells (Fig 2D, lanes 2 and 3, respectively). Therefore, SYK is unlikely to be a target of BAY61-3606 in AD169 infected HFF cells.

As BAY61-3606 is a kinase inhibitor we also investigated the possibility that the drug can inhibit the virally encoded kinase UL97. The mobility of UL97 from AD169 infected HFF cells treated with either DMSO, BAY61-3606 or the UL97 kinase inhibitor maribavir [25] in a polyacrylamide gel was assayed using western blotting (Fig 2E). We found that UL97 in AD169 infected cells treated with maribavir (Fig 2E, lane 4) had a molecular weight less than UL97 in infected cells treated with either DMSO or BAY61-3606 (Fig 2E, lanes 2 and 3, respectively). As maribavir can inhibit UL97 autophosphorylation [38], we propose that while maribavir can inhibit UL97 phosphorylation, resulting in the detection of low molecular weight unphosphorylated UL97, the presence of neither DMSO nor BAY61-3606 affected the protein kinase activity of UL97. As UL97 is produced late in HCMV replication, the low levels of UL97 in HCMV infected cells treated with either maribavir or BAY61-3606 compared to infected cells treated with DMSO, is likely due to inhibition of immediate-early protein production by these drugs (Fig 1 and [39]).

### Inhibition of IKKα kinase activity by BAY61-3606

**Fig 3:**
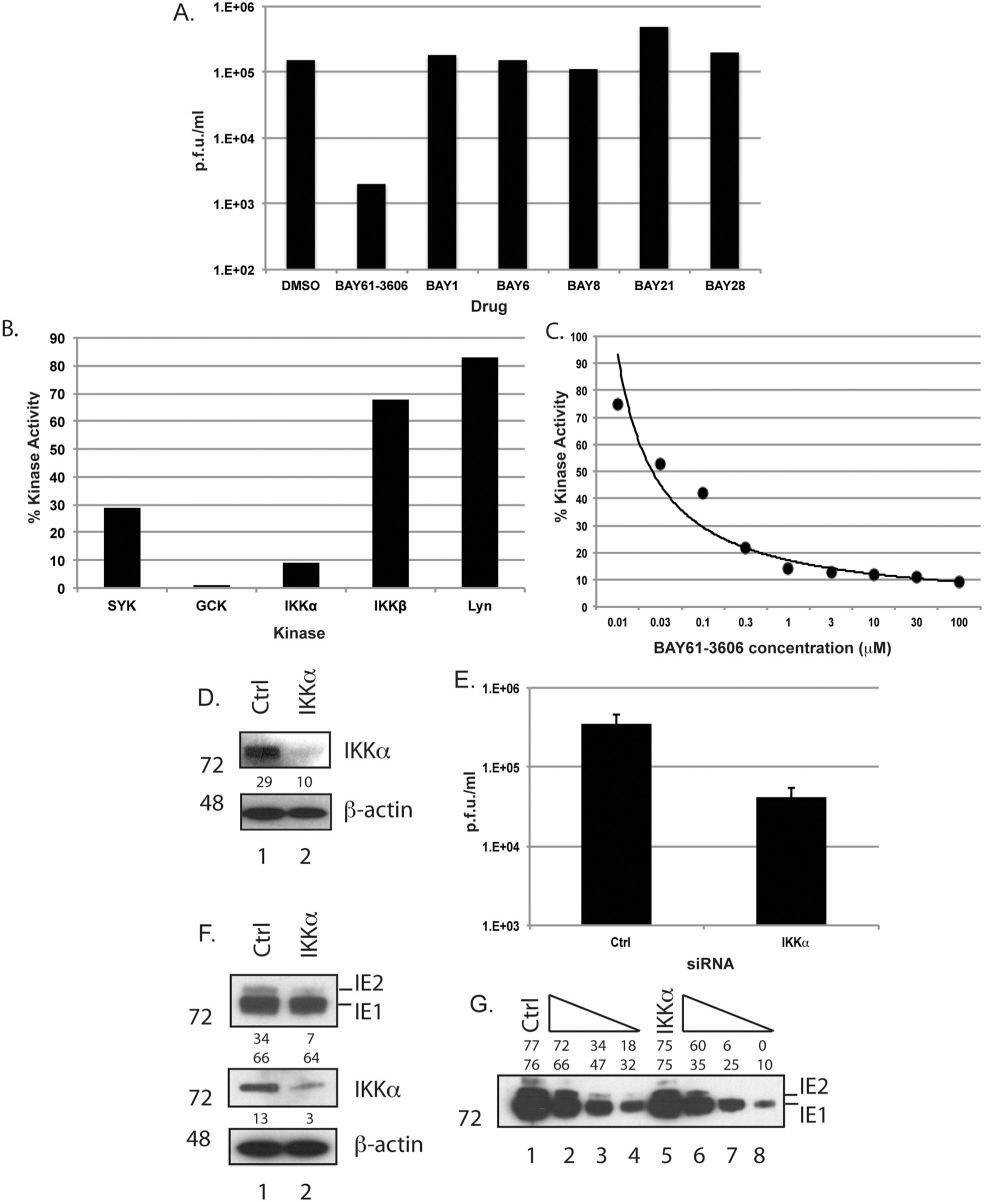
Identification of IKKα as a target of BAY61-3606. (A) Inhibition of AD169 replication by BAY compounds. HFF cells were infected with AD169 at an MOI of 1 then treated with 1μM of the drug indicated in the figure or the equivalent volume of DMSO. Viral supernatants were harvested at 96 h.p.i. and viral titre (p.f.u./ml) was determined. (B and C) In vitro kinase assays in the presence of BAY61-3606. The ability of 10 μM BAY61-3606 (Fig 3B) or a range of BAY61-3606 concentrations (Fig 3C) to inhibit the kinase activity of each of the indicated kinase proteins was assayed. Each data point in each figure represents the percentage kinase activity in the presence of drug compared to DMSO treated controls. Each data point shows the mean value of two experiments. (D-G) Analysis of HFF treated with siRNA. HFF cells were treated with either Crtl or IKKα siRNA. After 72 hours incubation with siRNA cell lysates were prepared for western blotting (Fig 3D) or infected with 1x10^5^ p.f.u. of HCMV to analyze virus replication by virus titration (Fig 3E). The data in Fig 3E is represented as p.f.u./ml at 96 h.p.i. and shows the mean and standard deviation of 3 experiments. Also, cell lysates from siRNA treated cells infected AD169 were prepared for western blotting 24 h.p.i. (Fig 3F). In Fig 3G, samples from lanes 1 and 5 of Fig 3E were diluted in a 2-fold series (lanes 2–4 and 6–8, respectively). The siRNA used in each case is indicated in each panel. In Fig 3D, 3F and 3G proteins recognized by the antibodies used in each experiment are indicated to the right of each figure and the positions of molecular weight markers (kDa) are indicated to the left of each figure. Band intensities are expressed in arbitrary units above or below each panel.

One of our laboratories has previously examined the ability of BAY61-3606 to inhibit a broad range of cellular kinases [22]. We reassessed this data and noted that the greatest inhibitory effect of BAY61-3606 was directed against the cellular kinase protein IKKα. Furthermore, we observed that a series of drugs structurally related to BAY61-3606 that do not inhibit IKKα (BAY1, BAY6, BAY8, BAY21 and BAY28 [22]) had no effect on AD169 replication (Fig 3A). Therefore, we hypothesized that IKKα may be required for productive AD169 replication.

To confirm BAY61-3606 is an inhibitor of IKKα we used *in vitro* kinase assays to assess the ability of BAY61-3606 to inhibit the kinase activity of a number of cellular kinases, including IKKα. We observed that BAY61-3606 is a potent inhibitor of SYK, GCK and IKKα, but not IKKβ or LYN, a kinase functionally unrelated to IKKα (Fig 3B). We also quantified the ability of BAY61-3606 to inhibit IKKα by measuring kinase activity in *in vitro* kinase assays over a range of drug concentrations (Fig 3C). We found that BAY61-3606 could inhibit IKKα kinase activity with a sub-micromolar 50% inhibitory concentration (IC50) (0.045 μM), indicating that BAY61-3606 is a potent IKKα kinase inhibitor.

**Fig 4:**
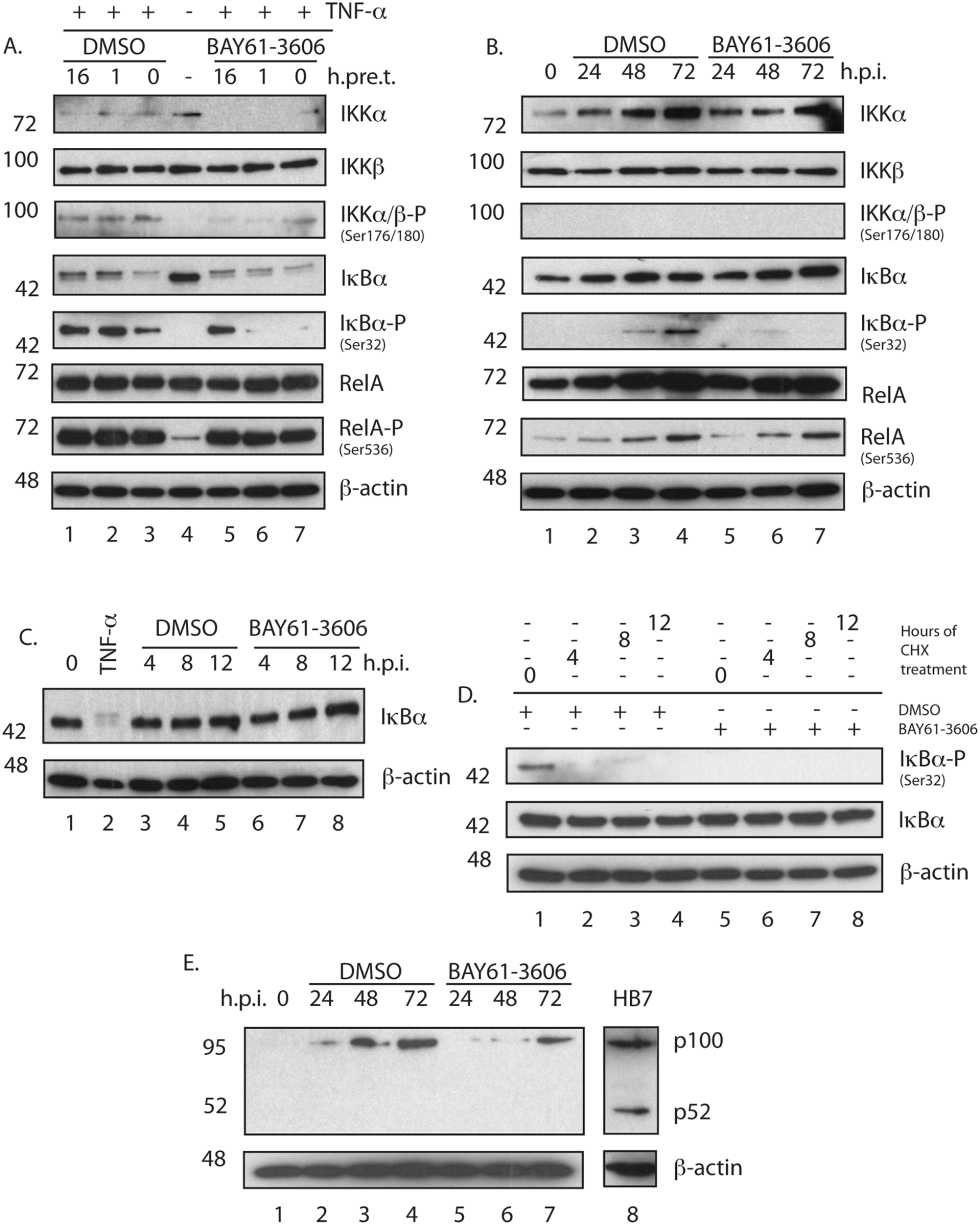
Investigation of canonical and non-canonical NF-κB signaling. (A) Canonical NF-κB signaling in uninfected HFF cells treated with TNF-α. Lysates of HFF cells prepared for western blotting after pre-treatment with either 1μM BAY61-3606 or the equivalent volume of DMSO and then treated with 10 ng/ml TNF-α for 5 mins. The number of hours pre-treatment (h.pre.t.) with DMSO or BAY61-3606 is indicated above the Fig 4A. Where cells were simultaneously treated with TNF-α and either DMSO or BAY61-3606 is indicated as 0 h.p.t. (B) Canonical NF-κB signaling in uninfected and infected HFF cells. HFF cells were uninfected or infected with AD169 at an MOI of 1, then treated with either 1μM BAY61-3606 or the equivalent volume of DMSO. Cell lysates were prepared for western blotting at the time points (hours post infection (h.p.i.)) indicated above the figure. Uninfected cells harvested at the time of infection are shown as 0 h.p.i.. (C) Analysis of IκBα degradation at early time points. HFF cells were uninfected (0 h.p.i.) (lane 1), uninfected and treated with 10 ng/ml TNF-α for 5 minutes (lane 2), or infected with AD169 at an MOI of 1, then treated with either 1μM BAY61-3606 or the equivalent volume of DMSO (lanes 6–8 and 3–5, respectively). Cell lysates were prepared for western blotting at the time points (hours post infection (h.p.i.)) indicated above the figure. (D) Analysis of cycloheximide treatment on IκBα degradation. HFF cells were infected with AD169 at an MOI of 1 then treated with either 1μM BAY61-3606 or the equivalent volume of DMSO for 72 hours. At 72 h.p.i. cell lysates were prepared for western blotting after treatment with 100 μg/ml cycloheximide for the time points (hours post treatment (h.po.t.)) indicated above the figure. (E) Non-canonical NF-κB signaling in uninfected and infected HFF cells. HFF cells were uninfected or infected with AD169 at an MOI of 1, then treated with either 1μM BAY61-3606 or the equivalent volume of DMSO. Cell lysates were prepared for western blotting at the time points (hours post infection (h.p.i.)) indicated above the figure. Uninfected cells harvested at the time of infection are shown as 0 h.p.i.. An equivalent volume of lysate from the EBV infected B cell line HB7 was also analyzed. Where indicated, uninfected cells harvested at the time of infection are shown as 0 h.p.i.. In each figure proteins recognized by the antibodies used in each experiment are indicated to the right of each figure. The positions of molecular weight markers (kDa) are indicated to the left of each figure.

To investigate the role of IKKα in HCMV replication, we treated HFF cells with either Ctrl siRNA or siRNA targeting *IKKα* mRNA (IKKα siRNA) and assayed either the presence of IKKα protein using western blotting or AD169 replication (Fig 3D and 3E, respectively). We observed that treatment of HFF cells with IKKα siRNA (Fig 3D, lane 2 and Fig 4E) notably depleted IKKα and lowered AD169 production by 3-fold and 10-fold, respectively, compared to HFF cells treated with Ctrl siRNA (Fig 3D, lane 1 and Fig 3E).

To complement these findings, we used western blotting to assay production of immediate-early proteins IE1 and IE2 in siRNA treated cells infected with HCMV (Fig 3F). At 24 h.p.i. we observed that accumulation of IE2 was approximately 4-fold lower in infected HFF cells treated with IKKα siRNA (Fig 3F, lane 2) compared to infected HFF cells treated with Ctrl siRNA (Fig 3F, lane 1). We also performed serial dilution of these samples and again assayed IE1 and IE2 protein levels by western blotting (Fig 3G). Here we observed approximately 3- to 5-fold reduction in accumulation of both IE1 and IE2 proteins in AD169 infected HFF cells treated with IKKα siRNA (Fig 3G, lanes 5–8) compared to AD169 infected HFF cells treated with Ctrl siRNA (Fig 3G, lanes 2–4) at various dilations. We observed similar phenotypes when infected cell lysates prepared at 48 and 72 h.p.i were analyzed for IE1 and IE2 levels by western blotting (data not shown).

Therefore, we confirm that BAY61-3606 is an inhibitor of IKKα and provide quantitative analysis of IKKα kinase inhibition by BAY61-3606. Similar phenotypes are observed upon treatment of AD169 infected HFF cells with BAY61-3606 and during AD169 replication in HFF cells treated with IKKα siRNA. Therefore, we propose that IKKα is a target of BAY61-3606 in AD169 infected HFF cells. Furthermore, treatment of AD169 infected cells with IKKα siRNA results in a greater decrease in viral replication compared to the observed decrease in immediate-early protein production. Therefore, inhibition of viral replication by IKKα siRNA is likely to inhibit the production or function of viral or cellular factors required for productive replication other than viral immediate-early proteins. This could include factors affected by the depletion of IKKα before infection.

### Canonical and non-canonical NF-κB signaling in uninfected and infected cells

To confirm that BAY61-3606 can act as an inhibitor of IKKα in HFF cells we assayed the ability of BAY61-3606 to inhibit canonical NF-κB signaling stimulated by TNF-α treatment in uninfected HFF cells (Fig 4A). HFF cells were untreated (Fig 4A, lane 4) or pretreated for 16 hours or 1 hour with either DMSO or BAY61-3606 before TNF-α treatment (Fig 4A, lanes 1–2 and lanes 5–6, respectively) or treated with either DMSO or BAY61-3606 at the time of TNF-α treatment (Fig 4A, lanes 3 and 7, respectively). After treatment of cells, cell lysates were prepared for western blotting and analyzed using antibodies that recognize components of the canonical NF-κB signaling pathway.

Treatment of cells with TNF-α and DMSO resulted in accumulation of IKKα/β-P(Ser176/180) and phosphorylation of the IKKα substrate IκBα (IκBα-P(Ser32)). Furthermore, IκBα, was present at low levels and increased in molecular weight, which is consistent with phosphorylation dependent activation of IκBα proteasomal degradation. Finally, consistent with activation of canonical NF-κB signaling, we observed increased levels RelA-P(Ser536). Treatment of cells with TNF-α and BAY61-3606 resulted in a loss of IKKα/β-P(Ser176/180), IκBα-P(Ser32), but no obvious difference in accumulation of IκBα. Also, treatment of cells with BAY61-3606 had no obvious effect on accumulation of RelA-P(Ser536). Therefore, the presence of BAY61-3606 is sufficient to inhibit phosphorylation of IκBα by IKKα in TNF-α treated HFF cells, although this is not sufficient to inhibit canonical NF-κB signaling under experimental conditions used here.

We hypothesized that treatment of HCMV infected cells with BAY61-3606 would lead to inhibition of canonical NF-κB signaling. Therefore, we assayed canonical NF-κB signaling in uninfected HFF cells (Fig 4B, lane 1) and HFF cells infected with AD169 and treated with either DMSO or BAY61-3606 (Fig 4B, lanes 2–4 and lanes 5–7, respectively). We found no IKKα-P(Ser176/180) in any sample. Moreover, although the presence of BAY61-3606, but not DMSO, decreased levels of IκBα-P(Ser32) we found no degradation of IκBα that would indicate canonical NF-κB signaling had occurred. Also, accumulation of RelA-P(Ser536) in AD169 infected cells treated with either DMSO or BAY61-3606 increased over time. Thus, while the presence of BAY61-3606 can inhibit phosphorylation of IκBα by IKKα in HCMV infected cells, lack of IKKα/β phosphorylation and lack of IκBα degradation indicates canonical NF-κB signaling does not occur. However, accumulation of RelA-P(Ser536) suggests that there may be transcriptional transactivation by RelA outside of canonical NF-κB signaling.

It has been reported that NF-κB signaling in HCMV infected cells occurs early in HCMV infection [6–8,12,40]. We, therefore, assayed degradation of IκBα by western blotting in uninfected HFF cells (Fig 4C, lane 1), uninfected HFF cells treated with TNF-α (Fig 4C, lane 2) or HFF cells infected with AD169 and treated with DMSO or BAY61-3606 from 4–24 h.p.i. (Fig 4C, lanes 3–5 and 6–8, respectively). We found degradation of IκBα in uninfected cells treated with TNF-α, but not in uninfected cells or infected cells treated with either DMSO or BAY61-3606.

As canonical NF-κB signaling results in production of IκBα [41], we also considered the possibility that canonical NF-κB signaling occurs in AD169 infected cells but *de novo* production of IκBα masks degradation of IκBα. We, therefore, investigated if IκBα was degraded in HFF cells infected with AD169 and treated with the protein synthesis inhibitor cycloheximide in the presence of either DMSO or BAY61-3606 (Fig 4D, lanes 2–4 and lanes 6–8, respectively). We did not observe degradation of IκBα under any condition. Therefore, while IκBα phosphorylation can occur in HCMV infected HFF cells a mechanism exists which prevents degradation of IκBα, which is essential for canonical NF-κB signaling. This would further indicate that canonical NF-κB signaling does not occur in this context and that IKKα is involved in a mechanism other than canonical NF-κB signaling that is required for productive HCMV replication.

IKKα is also required for non-canonical NF-κB signaling. Proteasomal processing of p100 to p52 is required for non-canonical NF-κB signaling. We used western blotting to assay levels of p100 and p52 in uninfected HFF cells (Fig 4E, lane 1), HFF cells infected with AD169 then treated with either DMSO or BAY61-3606 (Fig 4E, lanes 2–4 and 5–7, respectively), or the EBV positive B cell line HB7 (Fig 4E, lane 8) in which non-canonical NF-κB signaling should occur [42]. Processing of p100 and p52 was observed in HB7 cells but not uninfected or infected HFF cells. Thus, consistent with a previous report [13], non-canonical NF-κB signaling does not appear to occur in AD169 infected cells. Therefore, IKKα is involved in a mechanism required for productive AD169 replication other than non-canonical NF-κB signaling.

### Requirement of IKKα for H3 serine 10 phosphorylation

**Fig 5:**
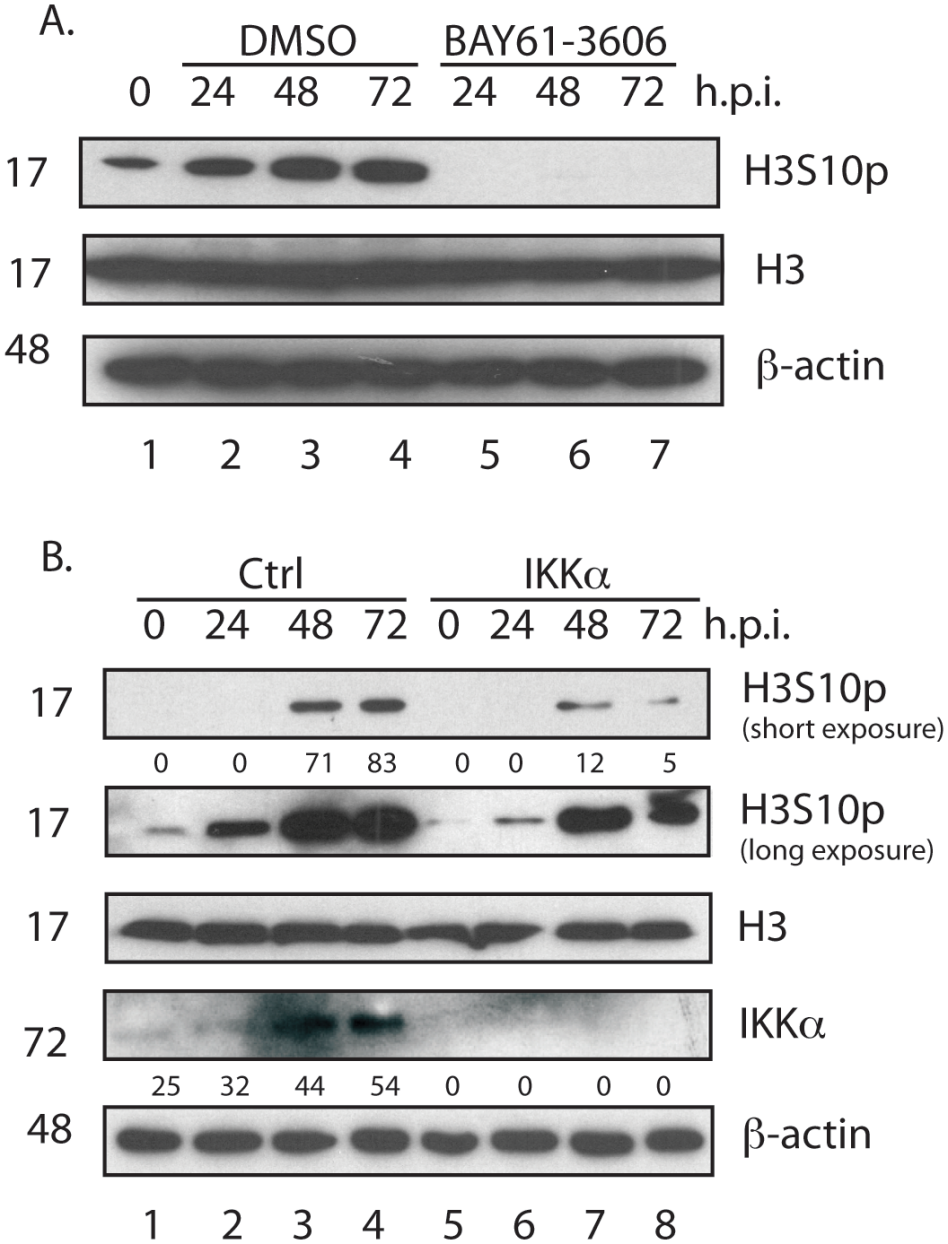
Analysis of H3 phosphorylation by IKKα. (A) Analysis of H3S10p in HCMV infected HFF cells treated with BAY61-3606. HFF cells were uninfected or infected with AD169 at an MOI of 1, then treated with either 1μM BAY61-3606 or the equivalent volume of DMSO. Cell lysates were prepared for western blotting at the time points (hours post infection (h.p.i.)) indicated above the figure. Uninfected cells harvested at the time of infection are shown as 0 h.p.i.. Short and a long exposures of the western blot to film to detect H3S10p are shown. (B) Analysis of H3S10p in infected cells treated with siRNA. HFF cells were treated with either Crtl or IKKα siRNA. After 72 hours incubation with siRNA cells were prepared for western blotting (0 h.p.i.) or infected with 1x10^5^ p.f.u. of AD169 and then prepared for western blotting at the time points (h.p.i.) indicated above the figure. The siRNA used are also indicated above the figure. In each figure proteins recognized by the antibodies used in each experiment are indicated to the right of each figure. The positions of molecular weight markers (kDa) are indicated to the left of each figure. In Fig 5B band intensities are expressed in arbitrary units below certain panels.

IKKα has been reported to phosphorylate histone H3 at serine residue 10 (H3S10p) [16,18–21]. Therefore, we used western blotting to investigate H3S10 phosphorylation in uninfected HFF cells (Fig 5A, lane 1) and HFF cells infected AD169 and treated with either DMSO or BAY61-3606 (Fig 5A, lanes 2–4 and 5–7, respectively). Histone H3 levels were equivalent between samples, however, we observed a notable decrease in H3S10p in infected cells treated with BAY61-3606 compared to infected cells treated with DMSO. Therefore, IKKα was likely required for H3S10 phosphorylation in AD169 infected HFF cells. Using western blotting we assayed H3S10 phosphorylation in uninfected and infected cells treated with either Ctrl siRNA or siRNA targeting *IKKα* (IKKα siRNA) (Fig 5B, lanes 1–4 and 5–8, respectively). Treatment of cells with IKKα siRNA depleted IKKα compared to cells treated with Ctrl siRNA. Levels of H3 were equivalent in all samples, however, we found less H3S10p in cells treated with IKKα siRNA compared to cells treated with Ctrl siRNA (approximately 6-fold and 16-fold decreases at 48 and 72 h.p.i., respectively). Therefore, IKKα is required for H3S10p in AD169 infected HFF cells and loss of H3S10p could result in inhibition of productive AD169 replication.

### The relationship between H3 phosphorylation, acetylation and methylation in HCMV infected cells

**Fig 6:**
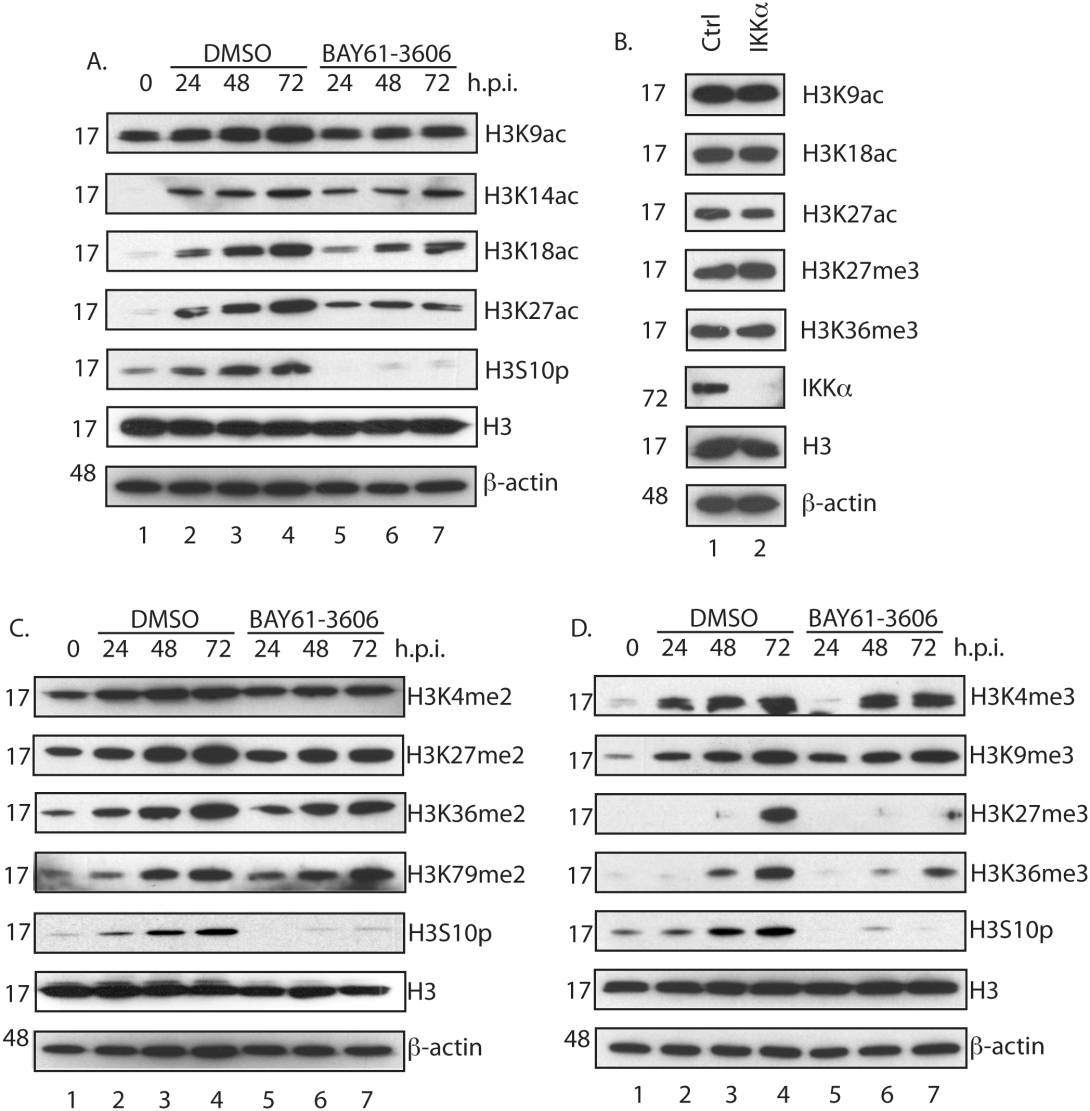
Investigation of histone H3 modifications in BAY61-3606 and siRNA treated cells. (A, C and D) Western blotting of HFF cells treated with BAY61-3606. HFF cells were uninfected or infected with AD169 at an MOI of 1, then treated with either 1μM BAY61-3606 or the equivalent volume of DMSO. Cell lysates were prepared for western blotting at the time points (hours post infection (h.p.i.)) indicated above the figure. Uninfected cells harvested at the time of infection are shown as 0 h.p.i.. (B) Western blotting of HFF cells treated with siRNA. HFF cells were treated with either Crtl or IKKα siRNA. After 72 hours incubation cells infected with 1x10^5^ p.f.u. of AD169 and then prepared for western blotting at 72 h.p.i.. The siRNA used is indicated above the figure. In each figure proteins recognized by the antibodies used in each experiment are indicated to the right of each figure. The positions of molecular weight markers (kDa) are indicated to the left of each figure.

Loss of H3S10p has been reported to result in loss of acetyl modifications of H3 [16,21] and at least one study indicates that loss of H3S10p reduces the total levels of H3K14ac in cells [16]. As the presence of H3K14ac on H3 is associated with transcriptional activation in HCMV infected cells [14], we hypothesized that loss of H3S10p might also lead to loss of H3K14ac, which would impact HCMV gene expression. We used western blotting to assay levels of H3, H3S10p and acetylation of H3 on a number of commonly studied H3 residues including K14 (H3K9ac, H3K14ac, H3K18ac, H3K27ac) in either uninfected HFF cells (Fig 6A, lane 1) or HFF cells infected with AD169 and treated with either DMSO or BAY61-3606 (Fig 6A, lanes 2–4 and 5–7, respectively). Levels of H3 were equivalent in all samples and treatment of HCMV infected cells with BAY61-3606, but not DMSO, lowered levels of H3S10p. There was little or no difference in accumulation of H3K14ac in infected cells treated with either DMSO or BAY61-3606. However, BAY61-3606 inhibited accumulation of H3K9ac, H3K18ac and H3K27ac.

To asses what differences in H3K9ac, H3K18ac, H3K27ac accumulation were attributable to inhibition of IKKα, we used western blotting to assay H3 acetylation levels in HFF cells treated with either Ctrl or IKKα siRNA and infected with AD169 (Fig 6B). While treatment of HFF cells with IKKα siRNA (Fig 6B, lane 2) decreased IKKα levels compared to cells treated with DMSO (Fig 6B, lane 1), we observed no obvious difference between H3K9ac, H3K18ac or H3K27ac.

Therefore, in AD169 infected cells treated with BAY61-3606 loss of H3S10p did not lead to loss of H3K14ac. However, the accumulation of BAY61-3606 decreased H3K9ac, H3K18ac or H3K27ac, which may contribute to the anti-HCMV effects of BAY61-3606. However, as loss of IKKα has no effect on H3K9ac, H3K18ac or H3K27ac, it is unlikely H3S10p is required to maintain these post-translational modifications of histone H3.

It is unknown what relationship, if any, there may be between H3S10p levels and levels of dimethylation (me2) and trimethylation (me3) of H3 in HCMV infected cells. Western blotting was used to assay H3, H3S10p, plus H3 dimethylation (H3K4me2, H3K27me2, H3K36me2, H3K79me2) or H3 trimethylation (H3K4me3, H3K9me3, H3K27me3, H3K36me3) in uninfected HFF cells (lane 1, Fig 6C and 6D, respectively) or HFF cells infected with AD169 in the presence of either DMSO or BAY61-3606 (lanes 2–4 and 5–7, Fig 6C and 6D, respectively). In both experiments H3 was equivalent in all samples and treatment of HCMV infected cells with BAY61-3606, but not DMSO, lowered levels of H3S10p. We found no obvious difference between DMSO and BAY61-3606 treatment in accumulation of any me2 modification examined. However, there was a decrease in H3K27me3 and H3K36me3 accumulation in cells treated with BAY61-3606 compared to DMSO treated cells. Also, we observed a difference in H3K4me3 accumulation in BAY61-3606 treated cells early, but not late in infection compared to DMSO treated cells. There was no obvious difference in H3K9me3 in infected cells treated with either DMSO or BAY61-3606.

We decided to focus on H3K27me3 and H3K36me3 modifications and again used siRNA to determine if IKKα is required for H3K27me3 and H3K36me3 modifications of H3 in AD169 infected cells (Fig 6B). We found no obvious difference when we compared H3K27me3 and H3K36me3. Therefore, in AD169 infected cells treated with BAY61-3606 inhibition of H3K27me3 and H3K36me3 accumulation could contribute to the anti-HCMV effects of BAY61-3606. Thus, BAY61-3606 can inhibit virus replication via pathways that do not involve IKKα. Furthermore, as loss of IKKα has no effect on H3K27me3 or H3K36me3 it is unlikely H3S10p is required to maintain these post-translational modifications of histone H3.

## Discussion

Our studies of BAY61-3606 targets and mechanism of action of reveal unexpected insights about the role of IKKα and intracellular signaling in HCMV infected cells. First, we considered a role for IKKα in NF-κB signaling in HCMV infected cells. The role of canonical NF-κB signaling in productive HCMV replication has been unclear as there are reports indicating canonical NF-κB signaling is either required [6], or not required [4,5], for productive HCMV replication. Our observations are consistent with previous reports that canonical NF-κB signaling is not required for productive AD169 replication [4,5] and we provide data indicating how canonical NF-κB signaling is compromised during AD169 infection. The lack of IKKα/β phosphorylation and lack of IκBα degradation indicates that canonical NF-κB signaling does not occur in our studies of AD169 infected cells. Inhibition of IKKα/β phosphorylation may be due to the presence of the viral protein UL26, which has recently been reported to antagonize IKKα/β phosphorylation [13]. However, we observe phosphorylation of IκBα, which is thought to require phosphorylation of IKKα/β. Therefore, it is possible that phosphorylation of IKKα/β is not required for IKKα to phosphorylate IκBα or phosphorylation of IKKα/β occurs at levels undetectable in our assays. Furthermore, we observe that although IκBα is phosphorylated in HCMV infected cells, degradation of IκBα does not occur. We propose this may be due to the presence of a viral antagonist of canonical NF-κB signaling encoded by HCMV. For example, the human herpesvirus varicella zoster virus encodes a protein (ORF61) that allows phosphorylation of IκBα, but inhibits IκBα degradation [43]. It is unknown if HCMV encodes a protein with a similar function.

We observe an increase in RelA-P(Ser536) over time in AD169 infected cells and suggest that this is the result of phosphorylation of RelA by kinases such as IKKβ [3], not the result of canonical NF-κB signaling *per se*. It has been reported that IKKβ is required for efficient productive AD169 replication [9,10]. It remains unknown how phosphorylation of RelA by IKKβ would facilitate productive HCMV replication but inhibition of IKKβ can impair immediate early gene expression [9,10]. This mechanism could be a confounding factor in the analysis of transcription from promoters responsive to canonical NF-κB signaling in AD169 infected cells.

It remains unclear in what context canonical NF-κB signaling is required for either viral or cellular transcription during productive HCMV replication as different HCMV strains and cell types have been used in different studies. It is likely that canonical NF-κB signaling is utilized by HCMV in only certain contexts. For example, canonical NF-κB signaling may be inhibited in fibroblasts by the presence of HCMV NF-κB modulators UL138 and UL144, to prevent anti-viral inflammatory responses [32]. As these proteins are encoded by low passage, but not high passage, HCMV strains [32] this would, in part, explain strain-dependent differences in NF-κB signaling. As Merlin(RCMV)1111) encodes the UL138 and UL144 it is likely Merlin(RCMV)1111) inhibits NF-κB signaling. Therefore, the inability of BAY61-3606 to inhibit Merlin(RCMV)1111) replication is consistent with a lack of NF-κB signaling in cells infected with Merlin(RCMV)1111). However, canonical NF-κB signaling is required during dissemination of HCMV from macrophage [44].

We propose H3S10 phosphorylation by IKKα is required for efficient AD169 viral and cellular gene expression and AD169 replication. H3S10 can also be phosphorylated by mitogen and stress kinase 1 (MSK1) [45] and H3S10 phosphorylation by MSK1 can be found at the MIEP during HCMV transcription in dendritic cells [46]. BAY61-3606 does not target MSK1 [22], but it is possible that the low levels of H3S10p seen in AD169 infected HFF cells treated with either BAY61-3606 or IKKα siRNA is the result of H3S10 phosphorylation by MSK1. Preliminary experiments from our laboratory indicate that H3S10 is phosphorylated in cells infected with Merlin(RCMV1111) (data not shown). Importantly, as we did not observe inhibition of Merlin(RCMV1111) replication by BAY61-3606 it is possible that IKKα is not required to phosphorylate H3S10 in Merlin(RCMV1111) infected cells. Rather, we speculate, MSK1 is responsible for H3S10 phosphorylation in Merlin(RCMV1111) infected cells. Therefore, it should be stressed that all histone modifications discussed here may be present in cells infected with Merlin(RCMV1111), but the cellular factors required to mediate those histone modifications differ between HCMV strains.

Our study suggests H3S10p affects both viral and cellular transcription during productive AD169 replication. It is possible that H3S10p is required for transcription from the MIEP as treatment of AD169 infected cells results in a decrease in immediate-early protein production. However, we propose that H3S10p may be of more importance for cellular transcription required for AD169 replication, as profound decreases in AD169 replication contrast with relatively modest decreases in immediate-early protein production in infected cells treated with either BAY61-3606 or IKKα siRNA. A significant future challenge will be to map the localization of H3S10p to viral and cellular promoters in HCMV infected cells and determine how treatment of infected cells with either BAY61-3606 or IKKα siRNA affects the presence of H3S10p at those promoters.

We initially hypothesized that the loss of H3S10p would lead to loss of H3K14ac. However, we found no obvious effect on H3K14ac in AD169 infected HFF cells treated with either BAY61-3606 or IKKα siRNA. It is unclear why there should be contrasting observations between previous reports [16] and data presented here, but it is possible that there is an as yet unrecognized mechanism in AD169 infected cells that ensures H3K14 acetylation takes place in the absence of H3S10p.

It remains unknown how loss of H3S10p in AD169 infected cells directly or indirectly affects virus replication. H3S10p has been used as a marker for mitosis. Thus, we considered the possibility that loss of H3S10p in infected cells treated with either BAY61-3606 or IKKα siRNA is due to cell cycle arrest. However, we exclude this possibility as BAY61-3606 does not inhibit a number of kinases that regulate mitosis [22] and we observed no obvious defect in cell division of uninfected HFF cells treated with either BAY61-3606 or IKKα siRNA (data not shown). Rather, we propose that loss of H3S10p could have pleiotropic effects in the infected cell. Firstly, the lack of H3S10p at viral or cellular promoters could impact upon the recruitment or removal of transcription factors from DNA. For example, the presence of H3S10p is required for binding of 14-3-3 transcription factors to chromatin in the presence of H3K9ac and H3K14ac [17] and H3S10p in the presence of H3K9me3 required to remove HP1, an inhibitory factor which is known to have a role in inhibiting HCMV transcription from the MIEP [47] and from promoters [48]. Also, H3S10p is required for chromatin condensation [49]. During productive HCMV replication chromatin condensation must occur for chromatin partitioning to take place [50,51]. This process condenses chromatin within the infected cell nucleus providing space for the development of viral replication compartments, within which viral genome replication occurs [51,52]. Thus, the loss of H3S10p may not allow the formation or function of viral replication compartments.

We also observed that treatment of infected HFF cells with BAY61-3606 inhibits accumulation of certain histone H3 acetylation and tri-methylation modifications not related to the presence of IKKα. The loss of these modifications, either alone or in combination, most likely contributes to inhibition of AD169 replication by BAY61-3606 via an IKKα-independent mechanism. It is unknown what proteins and pathways BAY61-3606 acts on to inhibit accumulation of the H3 acetylation and tri-methylation modifications discussed above. Also, the function of these histone H3 modifications in AD169 infected cells is unknown. Thus, the mapping of histone H3 acetylation and tri-methylation modifications affected by the presence of BAY61-3606 to viral and cellular promoters will illuminate how these histone modifications shape the transcriptional landscape required for virus replication. Moreover, BAY61-3606 may prove to be a useful chemical tool to identify kinase proteins required for histone H3 acetylation and tri-methylation modification in HCMV infected cells.

Our observation that BAY61-3606 is an inhibitor of IKKα has relevance beyond the study of HCMV replication. Firstly, the use of BAY61-3606 in experiments that implicate a role for SYK and GCK in intracellular signaling that also involves IKKα should be reconsidered. Secondly, BAY61-3606 is structurally unrelated to widely available inhibitors of IKKα. Thus, BAY61-3606 is a novel chemical scaffold from which novel IKKα inhibitors can be developed. These compounds could be used as chemical tools to study topics such as pathogen replication and the cellular response to inflammatory stimuli, plus have the potential to be used as novel anti-infective therapeutics.
